# Efavirenz poisoning in a 12 year old HIV negative African boy

**Published:** 2012-07-26

**Authors:** Rose Nazziwa, Moorine Sekadde, Francis Kanyike, Eric Wobudeya, Nicolette Nabukeera-Barungi

**Affiliations:** 1 Department of Paediatrics and Child Health, Makerere University, College of Health Sciences and Mulago National Referral Hospital, Uganda

**Keywords:** Efavirenz, poisoning, intoxication, adverse reaction, HIV

## Abstract

Efavirenz is an oral antiretroviral drug in the class of non nucleoside reverse transcriptase inhibitors. Toxicity at therapeutic doses has been documented but there is scarcity of data on presentation and management of Efavirenz overdose. We describe a case of Efavirenz poisoning in a 12-year old HIV Negative African boy with a very unique presentation after ingesting 3 grams of Efavirenz as a single dose. The most prominent problems were burning sensation in the throat immediately after ingestion then visual impairment one hour later then tremors, screaming at night and motor deficits in lower limbs for 5 days before admission. His medical history, physical exam and investigations revealed no other cause of his presentation other than the EFV. Unfortunately, it was not possible to do EFV levels. He was given supportive treatment and 10 days later he was completely well.

## Introduction

Efavirenz is an oral antiretroviral drug in the class of non nucleoside reverse transcriptase inhibitors [1–3]. Toxicity at therapeutic doses has been documented and it includes psychiatric symptoms like insomnia, confusion, memory loss, depression and more serious symptoms such as psychosis may occur in patients with compromised liver or kidney function [1–3]. Other side effects including skin rash, nausea, dizziness and headache may occur [1]. There is scarcity of data on presentation of Efavirenz overdose at very high levels. This child took 7.5 times the recommended dose for her weight of 35 kilograms and had a very unique presentation. We set out to describe a case of accidental Efavirenz poisoning in a 12 year old HIV negative child and to share experiences in its presentation and management.

## Observation

Clinical and laboratory evaluation was carried out on a previously well 12 year old boy who was admitted to Mulago Hospital in Kampala, Uganda. He presented with history of accidental ingestion of 3grams of Efavirenz (EFV) 5 days prior to admission. It was mistaken for Mebendazole tablets which were used for routine deworming. There were no suicidal intentions; and the EFV belonged to an aunt who had recently died of HIV/AIDS. He developed a burning sensation in the throat shortly after swallowing the medication which lasted several days. This was then followed by visual blurring which occurred within 1 hour of ingestion of medication. He progressed to develop tremors involving the upper limbs and marked lower limb weakness with inability to walk without support. He had a history of screaming at night but no convulsions or altered mentation. There was no history of fever or headache. The child had normal micturition and bowel habits. No yellow eyes were reported. These symptoms started soon after EFV ingestion and had never been experienced before by the patient.

Significant examination findings were in the neurological examination where the child had impaired vision to near objects but no obvious cranial nerve palsies. He had normal muscle tone, reduced power of grade 3 in both lower limbs and brisk knee reflexes. He had normal power in the upper limbs with no tremors. Sensation was intact in both upper and lower limbs. There were no signs of meningeal irritation. Other examination findings were essentially normal. Complete blood count, cerebrospinal fluid analysis, liver and renal function tests were all normal. HIV serology was negative.

A diagnosis of EFV poisoning was made and the child managed conservatively on maintenance intravenous fluids. He was hospitalized for 4 days and discharged when better. At discharge, he was advised to take plenty of oral fluids and come for regular follow up. Ten days following the medication ingestion, the child was noted to have normal vision, grade 4 power in the lower limbs and the screaming episodes had stopped. It took about 14 days for full recovery of symptoms.

## Discussion

We set out to describe the presentation of accidental EFV poisoning in a child. As far as we know, there is no data about what happens when a child takes 7.5 times the recommended dose of Efavirenz. This was accidental ingestion five 600 mg EFV tablets at once. It is unfortunate that we were unable to take the EFV plasma levels.

Burning sensation in throat was reported immediately after ingestion of the tablets. Studies have documented a strong and prolonged burning sensation in the mouth and throat when EFV is administered in aqueous liquid formulations [4, 5] .

Our patient presented with blurred vision within only one hour of ingestion. Although blurred vision has been described before as a side effect of EFV in a case report, it was noted after 3 months on treatment [6]. Other publications report it as mild but in this case it was quite distressing [1]. Other neurological manifestations in our patient included tremors and neuropathies which were severe enough to cause inability to walk. Muscle power was reduced and deep tendon reflexes were increased but sensation was intact. Another case report of accidental poisoning revealed similar exaggerated CNS effects [7]. The CNS symptoms seem to correlate with excessive EFV plasma levels of 59.4 mg/l [8]. Our patient had psychiatric effects including sleep disorders, hallucinations and nightmares. These have been widely reported as side effects of EFV affecting between 15 and 54% of patients [1, 2, 9].

Other reported psychiatric events include abnormal dreams, affective disorder, aggression, agitation, anxiety, confusion states, depressed mood, depression, euphoric mood, homicidal ideation, insomnia, irritability, major depression, mood swings, nervousness, nightmare, panic attack, phobia, post-traumatic stress disorder, sleep disorder, social phobia, stress symptoms and suicide attempt [10].

## Conclusion

The presentation of EFV poisoning in this child mainly consisted of exaggeration of known adverse effects and manifestation of some rarely reported effects. Despite the big dose of EFV, all the adverse effects spontaneously resolved within about two weeks.
